# Nutritional support for successful weaning in patients undergoing prolonged mechanical ventilation

**DOI:** 10.1038/s41598-022-15917-w

**Published:** 2022-07-14

**Authors:** Shih-Ching Lo, Kevin Sheng-Kai Ma, Yen-Ru Li, Zi-Yue Li, Cheng-Hung Lin, Hsing-Chun Lin, Shun-Fa Yang

**Affiliations:** 1 Institute of Medicine, Chung Shan Medical University, Taichung, 402 Taiwan, ROC; 2 Department of Nutrition, Chung Shan Medical University Hospital, Taichung, 402 Taiwan, ROC; 3 Department of Nutrition, Chung Shan Medical University, Taichung, 402 Taiwan, ROC; 4 Center for Global Health, Perelman School of Medicine, University of Pennsylvania, Philadelphia, PA 19104 USA; 5 Department of Epidemiology, Harvard T.H. Chan School of Public Health, Boston, MA 02115 USA; 6 Graduate Institute of Biomedical Electronics and Bioinformatics, National Taiwan University, Taipei, 11114 Taiwan, ROC; 7 Department of Management, Chung Shan Medical University Hospital, Taichung, 402 Taiwan, ROC; 8 Respiratory Care Center, Chung Shan Medical University Hospital, Taichung, 402 Taiwan, ROC; 9 Department of Information Technology, Chung Shan Medical University Hospital, Taichung, 402 Taiwan, ROC

**Keywords:** Nutrition disorders, Respiratory tract diseases, Predictive medicine, Nutrition

## Abstract

Successful weaning from ventilators not only improves the quality of life of patients, but also reduces medical expenses. The aim of this study was to explore the association between nutritional provision and successful ventilator weaning. In this retrospective study data from the Respiratory Care Center of Chung Shan Medical University Hospital between October, 2017 and July, 2019 on patient characteristics, amount of nutrition delivered, and clinical outcomes were retrieved. A total of 280 ventilated patients were enrolled and divided into successful extubation and failed weaning groups. There were 178 males (63.6%) and 102 females (36.4%) with a mean age of 67.3 ± 16.9 years. The successful extubation group consisted of patients who tended towards ideal body weight during the weaning process (BMI 23.9 ± 5.0 versus 22.7 ± 4.8 kg/m^2^, p < 0.001). Patients from both groups initially received the same nutritional intervention, while patients of successful extubation received significantly more calories and protein after weaning (23.8 ± 7.8 kcal versus 27.8 ± 9.1 kcal, p < 0.001 and 0.97 ± 0.36 g versus 1.14 ± 0.42 g, p < 0.001). Successful weaning was associated with higher survival rate (p = 0.016), shortened hospital stay (p = 0.001), and reduced medical costs (p < 0.001). Overall, nutritional support with high calories and protein was associated with the probability of successful ventilator weaning in patients undergoing prolonged mechanical ventilation. Adequate nutrition is a determinant of successful ventilator weaning.

## Introduction

Previous studies have demonstrated that several factors are independently associated with extubation success, such as age, Glasgow coma score (GCS)^1^, and swallowing attempts^2^. Factors that can lead to failed weaning include increased respiratory load, timing of tracheotomy, and decreased neuromuscular competence^3,4^. From the results of the Large observational study to UNderstand the Global impact of Severe Acute respiratory FailurE (LUNG SAFE) and WorldwidE AssessmeNt of Separation of pAtients From Ventilatory assistancE (WEAN SAFE) studies, potential independent predictors of discontinuation or withdrawal of mechanical ventilation (MV) are: patient characteristics (age^5–9^, body mass index (BMI)^10^, geo-economic area^11^), comorbidities^5–7,9,10,12^, in-hospital treatment^5^, and severity of illness^5–8^. Successful ventilator weaning not only improves the quality of life of patients, but also reduces medical expenses.

Adequate nutritional provision is important for survival and shortens discharge time in the critically ill population. Recently, several global nutritional guidelines for critically ill patients have been published to inform practitioners of the best evidence-based therapies. The American Society for Parenteral and Enteral Nutrition (ASPEN) and the Society of Critical Care Medicine (SCCM) guidelines include energy requirements of 25–30 kcal/kg/day and protein requirements of 1.2–2.0 g/kg/day^13^. The European Society for Parenteral and Enteral Nutrition (ESPEN) guidelines are for 20–25 kcal/kg/day and 1.3 g/kg protein equivalents per day^14^. Achieving at least 80% of the prescribed protein intake is optimal^15^. In contrast, a research study has shown that caloric intake/target of more than 64.6% in an intensive care unit (ICU) for 7 days is associated with significantly higher mortality, ICU-acquired infections^16–21^, duration of MV, and length of stay (LOS) in hospital^22^. Underfeeding and overfeeding may increase the risk of infection of ventilated patients^23^ and prolong ventilator weaning time^24^. Therefore, nutrition intervention plays a key role in critical care.

information systems can assist decision-makers in improving performance and patient care, with which through integrating business intelligence (BI) and hospital information systems real-time big data analytics is possible. In the present study, we developed predictive models of ventilator weaning using BI system and large datasets in a medical university hospital respiratory care center (RCC). The aim of this study is the monitoring of ventilated patient nutritional status to determine its association with successful ventilator weaning.

## Materials and methods

### Establishment of the BI system

Since 2005, our hospital has applied BI to the evaluation of big data to understand the outcomes of medical treatments and to perform real-time monitoring. As of July 2019, 28 items have been completed, including medical management, quality control, response to overall operating conditions, and timely warning messages to facilitate preventive measures.

This is a retrospective analysis of existing data from the Healthcare/Hospital Information System (HIS) of Chung Shan Medical University Hospital (CSMUH). This study integrated several units, such as the information center, RCC, and departments of management and nutrition. As a first step, the department of management of CSMUH was informed of the indicators and purposes of nutritional analysis for ventilator weaning. Following discussions with the information center, and according to the studied indicators, the SharePoint platform of HIS was automatically uploaded. For the second step, we worked with nutrition department staff to write programs to classify data and map comparison charts for simple and easy presentation of results, applying BI to real-time monitoring of ventilated patient nutritional status and collecting anthropometric measurements, food-related history, biochemical data, and patient history. For the third step, a hospital-wide campaign was conducted to promote this system. After establishment of the BI system and simplification of the analytical process, we explored the indicators and predictive models of successful weaning.

### Study population and setting

**Figure 1:**
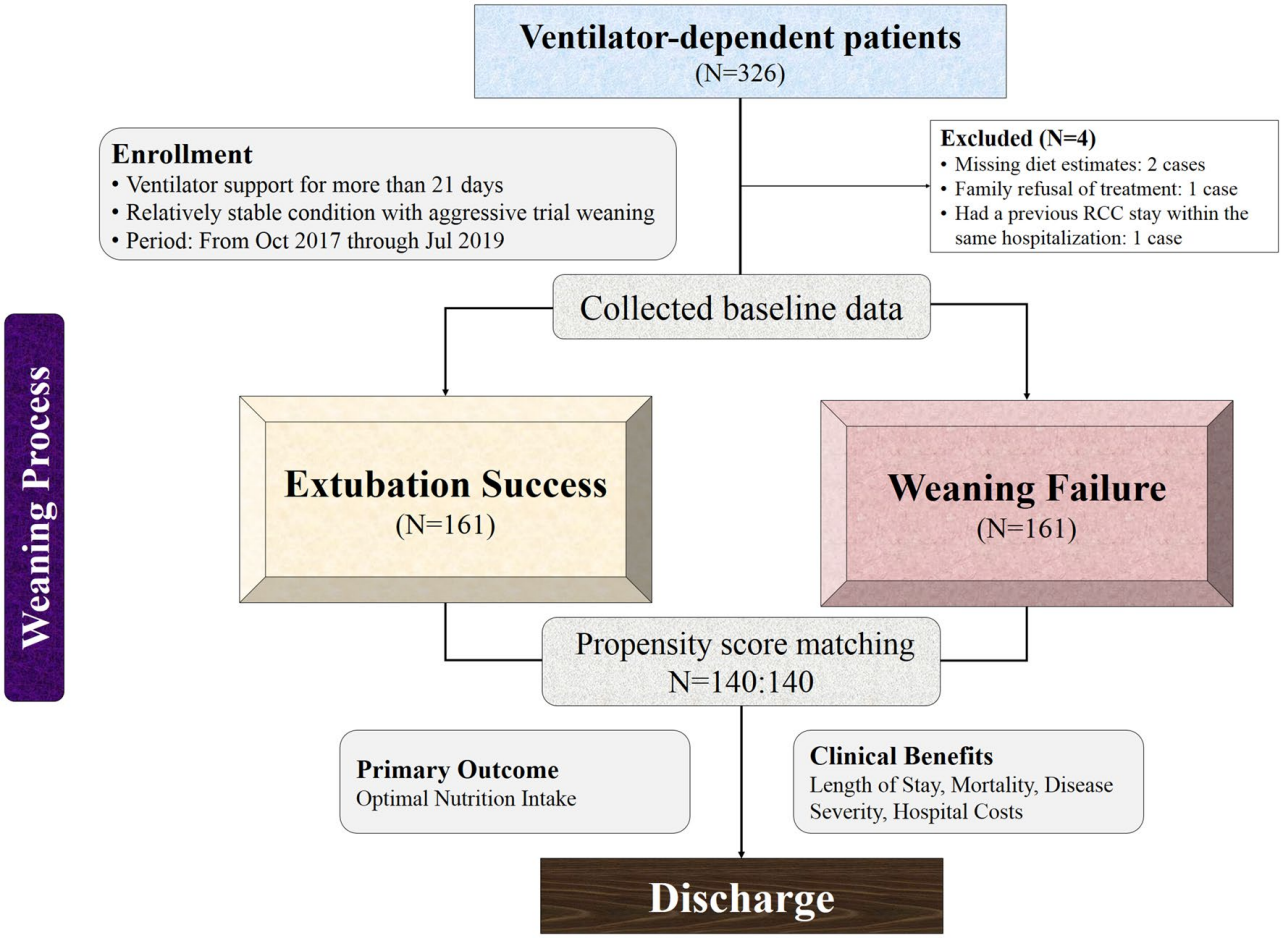
Enrollment flowchart and study protocol.

This observational cohort study was conducted to determine whether nutritional intake or other indicators are associated with successful extubation (Fig. 1). Participants had transferred to RCC and were studied from the time of ventilator-free protocol through the end of the weaning program at CSMUH from October 2017 to July 2019. Subjects were critically ill patients who were stable with prolonged MV-dependence (more than 21 days). The ventilator weaning protocol of CSMUH involves daily screening, followed by weaning parameter evaluation and process. Finally, T piece or continuous positive airway pressure (CPAP) is applied to achieve extubation (Supplemental Fig. 1). We recorded variables related to the use of BI during ventilator weaning. Patients in both groups received enteral nutrition (EN) support within 24 h, following assessment by a dietician. EN involved intermittent bag feeding with 5 meals per day. The total daily energy expenditure was estimated by a dietitian using the Harris-Benedict equation and adjusted for stress/active factors. Protein requirement was calculated as 0.9–1.5 g/kg based on patient condition and underlying diseases. Enteral feeding was suspended only if there were absolute contraindications, such as intestinal obstruction or hemodynamic instability^25^. Subjects were divided into successful extubation and failed weaning groups according to their actual status during the post-weaning period. Their basic information, nutritional intakes, laboratory data, and clinical outcomes were compared before admission to RCC and after transfer from RCC. Successful weaning was defined as removal of endotracheal tube and use of T-piece ventilation or CPAP^26^. Informed consent was waived by CSMUH for research projects in 2020 (CSH-2020-A-014) conducted according to the guidelines of the Declaration of Helsinki, with minimal risk to participants and approval from the Institutional Review Board (IRB) of CSMUH (IRB No. CS2-19144).

### Primary and secondary outcomes

We collected data on patient characteristics, nutritional intakes, and outcomes via BI system. Personal basic data, laboratory values during the experimental period, and nutritional data including types and amounts of nutrition prescribed and delivered (both calories and protein) were recorded during RCC stay. The primary outcomes of this study were the nutrition received during the optimal ventilator weaning program. Secondary outcomes included in-hospital mortality, medical costs, acute physiology and chronic health evaluation II (APACHE II) score, overall hospital LOS, and RCC LOS. We determined several predictors of successful extubation and survival.

### Statistical analysis

Normally distributed continuous variables are presented as means ± standard deviation (SD), while categorical variables are presented as numbers and proportions. National Health Insurance (NHI) premium and laboratory data are skewed and described using medians and interquartile ranges (IQRs). For hospital cost and biochemical data in non-normal distribution, we performed Mann–Whitney U test to compare differences between the groups. Two-sided p values of < 0.05 were considered significant. The Pearson chi-square test was used for categorical variables and the independent t test for continuous variables. The p values are from paired t test of continuous variables for within-group comparisons. Propensity score matching^27–34^ was based on gender, age, height, disease categories, initial weight, initial BMI, initial energy delivery, and initial protein intake. The propensity score was a probability that was estimated based on logistic regression. The binary variable was successful extubation and failed weaning. Every subject would create a propensity score between zero to one. Successful extubation group was matched to failed weaning group via 8-digit decimal point match of the propensity score. In the case of no match, 7-digit decimal point match was attempted by using nearest neighbor matching. The algorithm was allowed to proceed until no further matches could be made^35^. Survival was compared using log-rank tests and presented as Kaplan–Meier curves. We used post-hoc analysis to estimate the statistical power. Effect size was 0.26, alpha error was 0.05, group number was 140, and post-hoc power was 0.63. All analyses were performed using PASW Statistics 18, version 18.0.0 (formerly SPSS Statistics).

## Results

As shown in Fig. 1, a total of 326 ventilator-dependent patients were admitted to RCC and assessed for eligibility during the study period. Four patients were excluded, 2 due to missing EN data, 1 due to family’s refusal of treatment by a specialist physician, and 1 due to refractory transfer to ICU within the same hospitalization. Finally, 322 patients were enrolled in this study. Each group consisted of 161 patients. By matching the propensity score, a better balance in the heterogeneity between groups was achieved^35^. Finally, each group consisted of at least 140 patients. The parameters mentioned above were analyzed before and after the weaning process. Baseline characteristics of patients are shown in Table 1. There were no baseline differences in age, height, gender, BMI, or nutritional provision (both daily caloric and total protein intakes). Moreover, most of the laboratory data did not differ at baseline. Notable exceptions were significantly higher hemoglobin and phosphorus and lower blood urea nitrogen (BUN) and C-reactive protein (CRP).

**Table 1:** Baseline characteristics of enrolled patients.

Group	Successful extubation		Failed weaning		p value
	Number		Numer		
**Gender (%)**					0.267
Female	48	34.3%	54	38.6%	
Male	92	65.7%	86	61.4%	
Age	140	65.7 ± 17.9	140	68.9 ± 15.8	0.164
Height (cm)	140	162.3 ± 9.4	140	161.6 ± 9.1	0.458
**Studied group (%)**					0.141
Medical	69	49.3%	79	56.4%	
Surgical	71	50.7%	61	43.6%	
**Subspecialty (%)**					0.031*
Nephrology	16	11.4%	10	7.1%	
Chest/chest surgery	31	22.1%	38	27.1%	
Neurology/neurosurgery	64	45.7%	50	35.7%	
Gastroenterology	10	7.1%	6	4.3%	
Cardiology/cardiovascular surgery	10	7.1%	10	7.1%	
General surgery	4	2.9%	6	4.3%	
Hematology and oncology	5	3.6%	20	14.3%	
**Initial primary data**					
Weight (kg)	140	63.2 ± 14.7	140	60.6 ± 12.2	0.260
BMI (kg/m^2^)	140	23.9 ± 4.96	140	23.2 ± 4.15	0.256
APACHE II score	140	18.3 ± 3.6	140	19.9 ± 4.6	0.005**
Energy delivery (kcal/kg/day)	140	23.8 ± 7.8	140	24.5 ± 7.9	0.092
Protein intake (g/kg/day)	140	0.97 ± 0.36	140	1.01 ± 0.37	0.096
**Initial laboratory values**					
Albumin (g/dl)	134	3.16 ± 0.60	128	3.04 ± 0.61	0.193
Prealbumin(mg/dl)	50	19.7 ± 7.32	43	17.8 ± 7.87	0.309
Hemoglobin (g/dl)	123	10.7 ± 2.26	133	9.8 ± 1.87	0.011*
BUN (mg/dl)	135	34.0 ± 27.9	134	43.6 ± 36.0	0.011*
Creatinine (mg/dl)	138	1.72 ± 2.24	140	1.80 ± 1.76	0.161
Potassium (mmol/l)	138	4.10 ± 0.58	140	4.12 ± 0.74	0.840
Calcium (mg/dl)	68	8.25 ± 0.78	77	8.54 ± 0.96	0.136
Magnesium (mg/dl)	62	2.29 ± 0.46	57	2.35 ± 0.45	0.626
Phosphorus (mg/dl)	64	5.37 ± 2.09	56	3.47 ± 1.92	0.013*
CRP (mg/dl)	138	4.85 ± 5.27	138	7.06 ± 6.82	0.004**

Continuous data are expressed as mean ± SD and categorical data are expressed as n (%). The p values are from Pearson chi-square test for categorical variables and from independent t test for continuous variables. The p values are from the Mann–Whitney U test for laboratory data. *p values < 0.05 and **p values < 0.01 are considered statistically significant and extremely significant, respectively.

*BMI* Body Mass Index, *APACHE II score* Acute physiology assessment and chronic health evaluation II score, *BUN* blood urea nitrogen, *CRP* high sensitivity C reactive protein.

### Identifying at risk populations

The underlying diseases of ventilated patients were identified. There were significant differences in the successful extubation group versus the failed weaning group in terms of subspecialty (p = 0.031). Successful extubation group was mostly made up of surgical patients (50.7%) while failed weaning group was mostly made up of non-surgical patients (56.4%). Furthermore, a higher proportion of patients in the successful extubation group had neurological problems (45.7% vs 35.7%). There was a higher percentage of cancer patients in the failed weaning group (14.3% vs 3.6%), even though there were no baseline differences in cancer type^36–38^, nutritional status, or nutritional provision (data not shown).

### Primary outcome analysis

Initially, nutrition intervention was the same for successful extubation and failed weaning groups (Table 1, 23.8 ± 7.8 vs 24.5 ± 7.9 kcal/kg/day, p = 0.092 and 0.97 ± 0.36 vs 1.01 ± 0.37 g/kg/day, p = 0.096). However, during ventilator weaning, the successful extubation group received more calories and protein (Table 2, 23.8 ± 7.8 vs 27.8 ± 9.1 kcal/kg/day, p < 0.001 and 0.97 ± 0.36 vs 1.14 ± 0.42 g/kg/day, p < 0.001). There were no significant differences in caloric intake during weaning (Table 2, 24.5 ± 7.9 vs 25.6 ± 7.5 kcal/kg/day, p = 0.051 and 1.01 ± 0.37 vs 1.03 ± 0.36 g/kg/day, p = 0.348) or post-weaning (Table 3, 27.8 ± 9.1 vs 25.6 ± 7.5 kcal/kg/day, p = 0.199). After matching the propensity score, initial body weight of the successful extubation group did not significantly differ from that of the failed weaning group (Table 1, 63.2 ± 14.7 vs 60.0 ± 12.2 kg, p = 0.26). During the ventilator weaning process, the successful extubation group lost a significant amount of body weight. No body weight change was observed in the failed weaning group (Table 2, 63.2 ± 14.7 vs 59.5 ± 14.5 kg, p < 0.001 and 60.6 ± 12.2 vs 60.2 ± 11.6 kg, p = 0.284). In the post-weaning phase, there were no significant differences between the groups (Table 3, 59.9 ± 14.5 vs 60.2 ± 11.6 kg, p = 0.665).

**Table 2:** Clinical parameters during weaning from mechanical ventilation.

Group	Successful extubation		p-value	Failed weaning		p-value
Timeline	Initiation	Termination		Initiation	Termination	
Weight (kg)	63.2 ± 14.7	59.9 ± 14.5	< 0.001**	60.6 ± 12.2	60.2 ± 11.6	0.284
BMI (kg/m2)	23.9 ± 5.0	22.7 ± 4.8	< 0.001**	23.2 ± 4.2	23.0 ± 3.8	0.281
APACHE II score	18.3 ± 3.6	10.1 ± 6.8	< 0.001**	19.9 ± 4.6	20.6 ± 7.8	0.436
Caloric intake (kcal/kg/day)	23.8 ± 7.8	27.8 ± 9.1	< 0.001**	24.5 ± 7.9	25.6 ± 7.5	0.051
Protein intake (g/kg/day)	0.97 ± 0.36	1.14 ± 0.42	< 0.001**	1.01 ± 0.37	1.03 ± 0.36	0.348
Albumin (g/dl)	3.16 ± 0.6	3.29 ± 0.6	0.001**	3.04 ± 0.6	3.09 ± 0.6	0.236
Prealbumin (mg/dl)	19.7 ± 7.3	21.6 ± 6.9	0.025*	17.8 ± 7.9	22.0 ± 8.1	0.062
Hemoglobin (g/dl)	10.7 ± 2.3	11.0 ± 2.2	0.028*	9.83 ± 1.9	9.62 ± 2.0	0.159
BUN (mg/dl)	34.0 ± 27.9	35.9 ± 35.4	0.369	43.6 ± 36.0	42.8 ± 35.4	0.747
Creatinine (mg/dl)	1.72 ± 2.2	1.69 ± 2.4	0.707	1.80 ± 1.8	1.73 ± 1.6	0.369
Potassium (mmol/l)	4.10 ± 0.58	4.19 ± 0.67	0.107	4.12 ± 0.7	4.21 ± 0.8	0.188
Calcium (mg/dl)	8.25 ± 0.78	8.31 ± 1.08	0.697	8.54 ± 1.0	8.56 ± 0.9	0.817
Magnesium (mg/dl)	2.29 ± 0.46	2.30 ± 0.44	0.869	2.35 ± 0.4	2.29 ± 0.6	0.287
Phosphorus (mg/dl)	5.37 ± 2.1	4.74 ± 2.3	0.078	3.47 ± 1.9	4.18 ± 2.2	0.309
CRP (mg/dl)	4.85 ± 5.3	3.59 ± 5.1	0.006**	7.06 ± 6.8	6.68 ± 7.2	0.439

Continuous data are expressed as mean ± SD. The p values are from paired t test for continuous variables for within-group comparisons. *The p value < 0.05 and **p value < 0.01 are considered statistically significant and extremely significant, respectively.

*BMI* Body Mass Index, *APACHE II score* Acute physiology assessment and chronic health evaluation II score, *BUN* blood urea nitrogen, *CRP* high sensitivity C reactive protein.

**Table 3:** Comparisons of total outcomes between groups.

Group	Successful extubation		Failed weaning		p value
	Number		Number		
**Post-weaning period**					
Weight (kg)	140	59.9 ± 14.5	140	60.2 ± 11.6	0.665
BMI (kg/m^2^)	140	22.7 ± 4.84	140	23.0 ± 3.83	0.395
APACHE II score	79	10.1 ± 6.75	64	20.6 ± 7.80	< 0.001**
Energy delivery (kcal/kg/day)	140	27.8 ± 9.1	140	25.6 ± 7.5	0.199
Protein intake (g/kg/day)	140	1.14 ± 0.42	140	1.03 ± 0.36	0.140
**Clinical benefits**					
RCC LOS (day)	140	19.9 ± 10.0	28.5 ± 14.5		< 0.001**
Length of stay (day)	140	48.3 ± 19.5	56.3 ± 26.9		0.001**
NHI premium cost (USD)	138	$18,555 (13,498–27,175)		$26,312 (17,599–32,824)	< 0.001**
Mortality (%)	17	12.1%	36	25.7%	0.016*
**Post-weaning lab data**					
Albumin (g/dl)	108	3.29 ± 0.6	100	3.09 ± 0.6	0.026*
Prealbumin (mg/dl)	44	21.6 ± 6.9	38	22.0 ± 8.1	0.970
Hemoglobin (g/dl)	125	11.0 ± 2.2	121	9.6 ± 2.0	< 0.001**
BUN (mg/dl)	129	35.9 ± 35.4	124	42.8 ± 35.4	0.027*
Creatinine (mg/dl)	134	1.69 ± 2.4	130	1.73 ± 1.6	0.102
Potassium (mmol/l)	137	4.19 ± 067	132	4.21 ± 0.83	0.569
Calcium (mg/dl)	37	8.31 ± 1.08	26	8.56 ± 0.91	0.255
Magnesium (mg/dl)	21	2.3 (1.9–2.4)	18	2.3 (2.0–2.6)	0.460
Phosphorus (mg/dl)	30	3.8 (3.1–6.0)	19	3.7 (3.2–5.2)	0.954
CRP (mg/dl)	136	1.57 (0.59–4.21)	125	4.41 (1.53–8.45)	< 0.001**

Continuous data are expressed as mean ± SD and p values are from independent t test. Categorical data are expressed as n (%) and the p values are from the Pearson chi-square test. NHI premium cost and laboratory values are presented as median (25th to 75th percentiles), with p values from the Mann–Whitney U test. Mortality rates were compared using log-rank tests for survival analysis. *p value < 0.05 and **p value < 0.01 are considered statistically significant and extremely significant, respectively.

*BMI* Body Mass Index, *APACHE II score* Acute physiology assessment and chronic health evaluation II score, *RCC LOS* Respiratory care center length of stay, *NHI* National Health Insurance, *BUN* blood urea nitrogen, *CRP* high sensitivity C reactive protein.

Only the successful extubation group demonstrated significantly reduced APACHE II scores during the weaning process (Table 2, from 18.3 ± 3.6 to 10.1 ± 6.8, p < 0.001), with no change in disease severity in the failed weaning group (Table 2, from 19.9 ± 4.6 to 20.6 ± 7.8, p = 0.436). Similar findings for serum albumin and CRP were observed (Table 2). Serum phosphorus levels significantly differed at baseline. However, there were no differences in serum phosphorus levels within groups (p = 0.078 and 0.309) or between groups (p = 0.954) at the end of the study period. There were significant differences in BUN at baseline and at the end of the study period (p = 0.011 and 0.027), but not during the weaning period (p = 0.369 and 0.747).

### Secondary outcomes and clinical benefits

**Figure 2:**
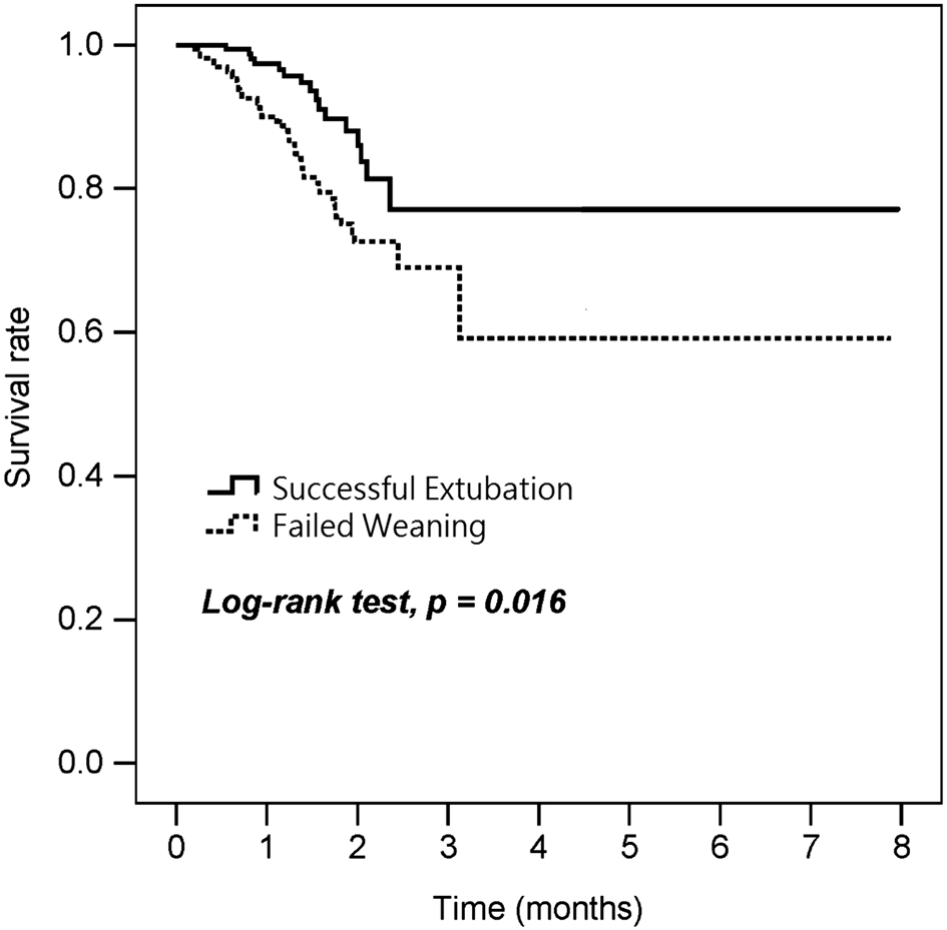
Survival curves for patients of successful extubation and patients of failed weaning.

The survival rate of the successful extubation group was significantly higher than that of the failed weaning group (Fig. 2, p = 0.016). As shown in Table 3, the successful extubation group had a mortality rate of 12.1% and the failed weaning group had a mortality rate of 25.7%. Furthermore, RCC LOS (Table 3, 19.9 ± 10.0 vs 28.5 ± 14.5 days, p < 0.001) and hospital LOS (Table 3, 48.3 ± 19.5 vs 56.3 ± 26.9 days, p = 0.001) were reduced. In the successful extubation group, medical costs were reduced by $5,475 per person on average when compared with the failed weaning group (Table 3, p < 0.001).

## Discussion

In this study we found that more near the target caloric intake, and protein delivery above 1.2 g/kg/day can improve nutrition for successful ventilator weaning. Optimizing nutrition for critically ill patients can improve their clinical outcomes^39–41^. ASPEN and SCCM guidelines include weight-based equations of 25–30 kcal/kg/day and recommended protein intakes of at least 1.2 g/kg/d^13^. This is consistent with our experimental results, which showed that reducing caloric and protein deficits is associated with successful ventilator weaning and significantly lower mortality (Fig. 2). In the FEED Trial, targeted energy and protein delivery were associated with attenuation of muscle loss and malnutrition at ICU discharge^42^. Higher caloric and protein deficits in the surgical intensive care unit are associated with lower discharge rates^43^. There is a significant and positive association between nutritional adequacy and 6-month survival in patients with higher Nutrition Risk in Critically ill (NUTRIC) score^44^.

According to the literature, patients with COPD or postoperative neuromuscular disease are at high risk of developing ventilator dependence^45^. A weaning protocol that combines respiratory parameters with neurologic measures leads to superior outcomes^46^. We identified the population with a high weaning success rate, i.e., neurosurgical patients with the capacity to undergo aggressive extubation therapy. However, poor outcomes and extubation failure have been demonstrated in medical patients who are elderly and have underlying chronic cardiac^47^–^50^ or respiratory disease^9^, which is consistent with our results. Similarly, a previous study did not show an improvement in the rates of successful ventilator weaning among cancer patients after the implementation of a resource-intensive weaning program^51^. It has also been demonstrated that APACHE II score is useful for predicting the outcome of weaning in ventilator patients^52^, consistent with our findings.

Intensive care unit-acquired weakness (ICU-AW) is a common complication with a clinically relevant impact on short- and long-term outcomes^53^. Initial body weight is a potential factor. Being mildly overweight is associated with successful weaning. Previous research has demonstrated that macronutrient deficit in the surgical intensive care unit is associated with worse in-hospital outcomes and lower rates of home discharge^43^, which is in line with the results of this study. Also consistent with our findings, correcting the serum phosphorus level has no effect on clinical outcome^54^ while increasing the hemoglobin level is positively associated with preferable weaning outcomes^55^. In this study, the hemoglobin level^56^ of the successful extubation group was higher than 10 g/dL^57^. Hyperinflammation induces elevated CRP level-related failure of weaning^58^. As shown on subgroup analysis in this study, this condition was more significant in the surgical patients than in the medical patients (Supplementary Table 1). Further studies are needed to clarify these results.

Of clinical relevance is that enteral feeding intolerance in mechanically ventilated critically ill patients is associated with malnutrition, fewer ventilator-free days (VFDs), longer LOS, and increased mortality^40^. From clinical outcomes (successful or failed weaning), we identified and assessed the influence of nutritional intervention. Recent guidelines have recommended the use of standardized protocols to reduce weaning duration and ICU LOS^59^. This study was based on our weaning practice. Higher caloric intake compared to lower caloric intake modifies the outcome and more protein delivery is recommended for nutritional support during ventilator weaning. We suggest 25–30 kcal/kg/day with > 1.2 g/kg protein for a better outcome.

In recent decades, there has been a growing body of research on artificial intelligence (AI) for precise diagnostics^60^. Various technical applications have been intensively researched to improve the accuracy of AI-facilitated diagnoses^61^. Moreover, through BI, deep learning has led to the development of predictive models based on large datasets^62^. The application of BI system to real-time monitoring of nutritional support provides more sophisticated information for developing ventilator weaning strategies. In addition, interactive visualization capabilities improve overall respiratory therapies and enhance efficiency.

As this was a retrospective observational study, there were some limitations. First of all, some baseline laboratory values differed between groups, including albumin, hemoglobin, phosphorus, BUN, and CRP. A downside of this study is that we did not collect data on the tracheostomy rate. However, the tracheostomy rate tends to be low in Taiwan due mainly to cultural factors, even though the “shared decision-making”^63^ protocol has been implemented. Second, we focused only on the ventilator weaning period during RCC stay, for which comprehensive data from admission, ICU, to post-acute care may augment our findings. Furthermore, as data on comorbidities were not collected, we were unable to explore their effects on the weaning process. Moreover, the number of cases was relatively small and the cases were localized. In future studies, data from multiple medical centers^64,65^ should be linked to validate the findings. That said, findings of this study revealed that the use of AI can greatly contribute to clinical medicine.

## Conclusions

Delivery of proteins and calories around the target calories intake allowed for preferable weaning outcomes in patients undergoing prolonged mechnical ventilation.

## Supplementary Information

Supplementary Information.

## Data Availability

The data sets generated and analyzed during the current study are available from the corresponding author upon reasonable request.
