# Supplementary material for: Nutritional support for successful weaning in patients undergoing prolonged mechanical ventilation
Source: Sci Rep. 2022 Jul 14;12:12044. doi: 10.1038/s41598-022-15917-w (PMC9283331; doi:10.1038/s41598-022-15917-w)
Supplement: Supplementary file 1 — Supplementary Information. [file 41598_2022_15917_MOESM1_ESM.docx]

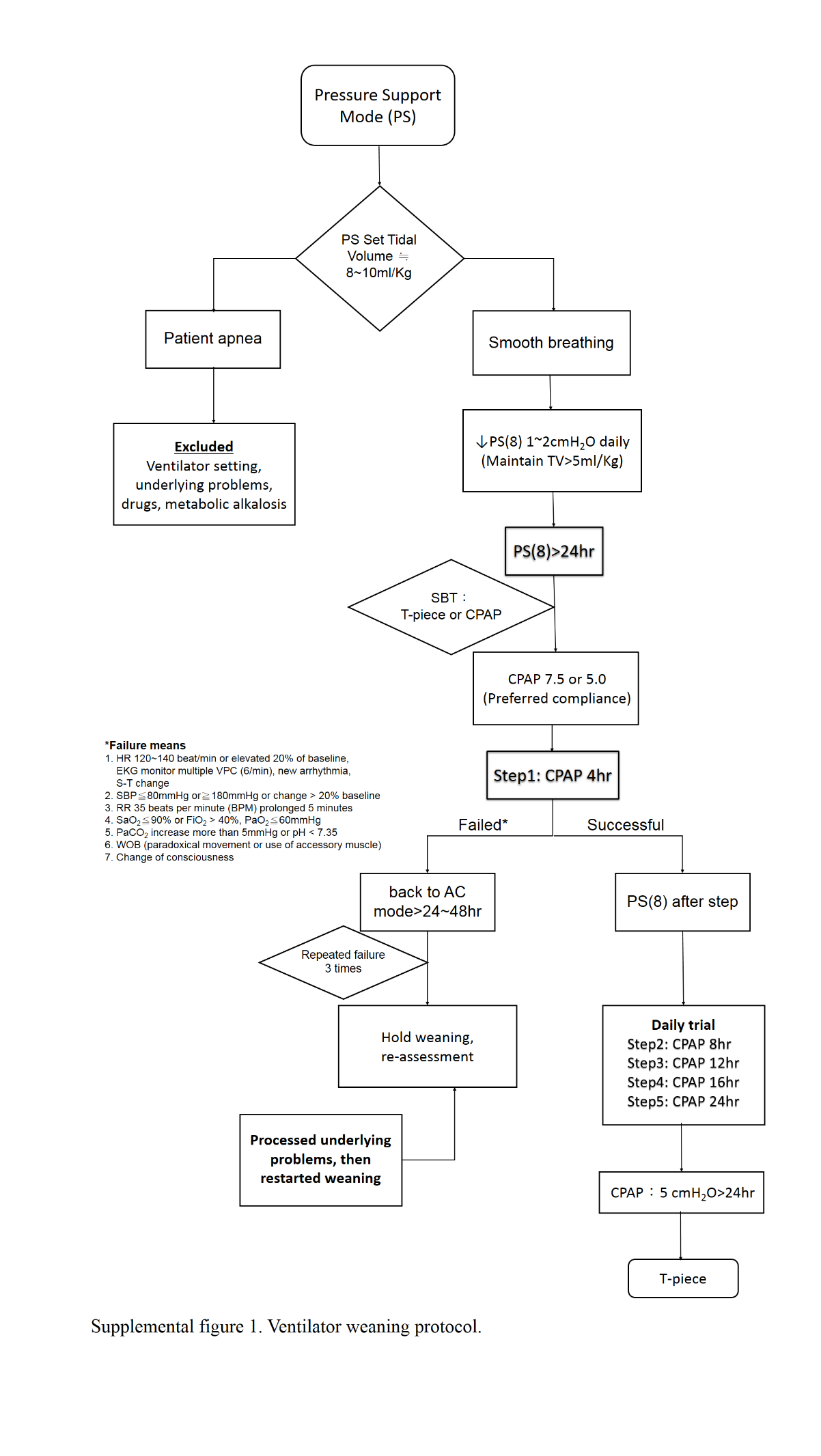


Supplemental figure 1. Ventilator weaning protocol.

Supplementary Table 1. Baseline characteristics of medical and surgical patients at enrollment

| **Group** | Medical | | **p-value** | Surgical | | **p-value** |
| --- | --- | --- | --- | --- | --- | --- |
|  | Success^(N=69)^ | Failure^(N=79)^ |  | Success^(N=71)^ | Failure^(N=61)^ |  |
| Weight _(kg)_ | 57.0 ±13.2 | 59.9 ± 12 | 0.212 | 62.8 ±15.2 | 60.7 ±11.2 | 0.620 |
| BMI _(kg/m_^2^_)_ | 21.93 ± 4.74 | 22.96 ± 4.05 | 0.148 | 23.20 ± 4.86 | 23.09 ± 3.56 | 0.866 |
| APACHE II score | 12.9 ± 7.5 | 21.1 ± 8.5 | <0.001** | 7.6 ± 4.9 | 20.0 ± 6.9 | <0.001** |
| Caloric intake_(kcal/kg/day)_ | 29.1 ± 9.75 | 25.3 ± 7.84 | 0.048* | 26.4 ± 8.24 | 26.1 ± 7.15 | 0.868 |
| Protein intake _(g/kg/day)_ | 1.16 ± 0.48 | 1.02 ± 0.36 | 0.222 | 1.12 ± 0.36 | 1.05 ± 0.37 | 0.422 |
| Albumin _(g/dl)_ | 3.18 ± 0.57 | 3.03 ± 0.69 | 0.348 | 3.40 ± 0.57 | 3.12 ± 0.50 | 0.013* |
| Prealbumin_(mg/dl)_ | 21.3 ± 7.2 | 22.5 ± 7.2 | 0.562 | 23.4 ± 6.7 | 21.9 ± 10.2 | 0.506 |
| Hemoglobin _(g/dl)_ | 10.38 ± 2.25 | 9.56 ± 2.05 | 0.026* | 11.58 ± 2.09 | 9.70 ± 2.04 | <0.001** |
| BUN _(mg/dl)_ | 47.5 ± 41.4 | 46.7 ± 35.4 | 0.735 | 24.8 ± 23.6 | 37.6 ± 35.0 | 0.015* |
| Creatinine _(mg/dl)_ | 2.16 ± 2.69 | 1.78 ± 1.58 | 0.890 | 1.26 ± 1.92 | 1.66 ± 1.67 | 0.101 |
| Potassium _(mmol/l)_ | 4.28 ± 0.75 | 4.16 ± 0.84 | 0.133 | 4.11 ± 0.57 | 4.26 ± 0.82 | 0.362 |
| Calcium _(mg/dl)_ | 8.45 ± 1.40 | 8.62 ± 0.91 | 0.426 | 8.35 ± 0.53 | 8.50 ± 0.94 | 0.304 |
| Magnesium _(mg/dl)_ | 2.36 ± 0.44 | 2.11 ± 0.22 | 0.101 | 2.05 ± 0.30 | 2.37 ± 0.68 | 0.111 |
| Phosphorus _(mg/dl)_ | 4.58 ± 2.29 | 4.03 ± 0.85 | 0.983 | 4.40 ± 1.97 | 4.54 ± 2.94 | 0.665 |
| HS C.R.P _(mg/dl)_ | 4.73 ± 6.16 | 6.51 ± 7.77 | 0.044* | 2.51 ± 3.67 | 6.81 ± 6.48 | <0.001** |

Continuous data are expressed as mean ± SD and p values are from independent t test and Mann-Whitney U test. * P values < 0.05 and ** p < 0.01are considered statistically significant and extremely significant differences, respectively.

**Abbreviations:** BMI- Body Mass Index, APACHE II score- Acute Physiology Assessment and Chronic Health Evaluation II score, BUN- Blood Urea Nitrogen, HS C.R.P- High Sensitivity C Reactive Protein.
